# Nonadhesive membranes: preparation and characterization of modified PHBHX membranes

**DOI:** 10.55730/1300-0527.3710

**Published:** 2024-11-20

**Authors:** Funda ALKAN, Murat DEMİRBİLEK, Oktay AYDIN, Berrak GÜMÜŞKAYA ÖCAL, Nelisa LAÇİN TÜRKOĞLU, Mustafa TÜRK, Mehmet Ali ONUR

**Affiliations:** 1 Bioengineering Division, Institute of Science, Hacettepe University, Ankara, Turkiye; 2 Department of Biology, Faculty of Arts and Sciences, Hacı Bayram Veli University, Ankara, Turkiye; 3 Department of General Surgery, Faculty of Medicine, Kırıkkale University, Kırıkkale, Turkiye; 4 Department of Medical Pathology, Surgical Medical Sciences, Faculty of Medicine, Yıldırım Beyazit University, Ankara, Turkiye; 5 Department of Molecular Biology and Genetics, Faculty of Arts and Sciences, Yıldız Teknik University, İstanbul, Turkiye; 6 Bioengineering Division, Engineering Faculty, Kırıkkale University, Kırıkkale, Turkiye; 7 Department of Biology, Faculty of Sciences, Hacettepe University, Ankara, Turkiye

**Keywords:** PHBHX, antiadhesive membrane, fatty acids, polyethylene glycol

## Abstract

After abdominal surgery, there is a possibility of adhesions between the abdominal organs and the abdominal wall. The adhesions can lead to problems such as chronic pain, intestinal blockage, and infertility. To prevent adhesion, antiadhesion patches can be used. In this study, poly hydroxybutyrate-co-hexanoate membranes were fabricated as antiadhesion patches and modified with either fatty acids or polyethylene glycol. The homogeneity and protein absorption of the membranes were assessed. The effects on blood coagulation factors were determined and the adhesion-proliferation properties of human fibroblast cells on the membranes were determined. The results show that myristic acid slightly increases surface free energy (40.7 ± 4.2 mN/m), decreases polar interaction (6.7 ± 0.7%), and has no effect on cell adhesion or proliferation at low concentrations, but does at high concentrations. Oleic acid slightly increases surface free energy (45.91 ± 4.8 mN/m), does not affect polar interaction (11.4 ± 0.9%), and increases cell proliferation at low concentrations. Both polyethylene glycol 400 and polyethylene glycol 8000 decrease cell adhesion and proliferation and do not change the surface free energy of membranes (39.6 ± 2.6 mN/m and 37.8 ± 1.8 mN/m, respectively), but decrease polar interaction (6.6 ± 0.3% and 5.1 ± 0.2%, respectively). In conclusion, the modified membrane is a good candidate for an antiadhesion patch for abdominal surgery.

## 1. Introduction

Adhesion of visceral organs to the abdominal wall is a problem in abdominal surgery. Chronic abdominal pain, bowel obstruction, and infertility are just some of the signs and symptoms of abdominal wall adhesions [1]. To prevent adhesion, antiadhesion patches can be used. Several properties of a patch are related to adhesion, including hydrophilicity, swelling ability, mechanical strength, surface charge, and chemical composition. Implantation may activate coagulation so that a fibrin layer forms on a patch. In a typical reaction of mesothelial tissue, mesothelial cells move toward a substance and wound site. Mast cells release histamines and other mediators, which increases vascular permeability and causes the formation of fibrin. There is an increase in both fibrinolytic activity and plasminogen activator inhibitor concentrations. Thus, fibrinous tissue forms on the patch and fibrin bands are formed. The fibrin band contains filamentous protein, leukocytes, erythrocytes, and platelets [2].

The occurrence of fibrin bands is primarily related to surface properties related to the electrokinetic potential, such as the charge of the patch material. When a biomaterial is immersed in a liquid, such as plasma, a reaction occurs at the solid–liquid interface. Due to the high dielectric coefficient of water, a negative charge accumulates on the surface of the material. The positively charged layer surrounding the surface is called the static layer. This layer is expected to neutralize the surface charge. However, since this is not completely possible, anions interacting with the surface form the secondary layer, which is also called the diffusion layer. This layer is relatively far from the surface. While the arrangement of ions in the stationary layer is quite regular, the ions in the diffusion layer are irregular, and the number of anions decreases farther from the surface. This layer is known as the Gouy–Chapman layer [3]. The potential difference between the negatively and positively charged layer on the surface of the material is called the electrokinetic (zeta) potential and is also used to describe the surface charge. As the thickness of the double layer increases, the capacitive property of the material increases [4]. When body fluid contacts a biomaterial surface, it behaves in two different ways; on hydrophobic surfaces, it has a relatively less dense water region and more hydrogen bonds, while on hydrophilic surfaces, it has a relatively dense water region and weaker intermolecular hydrogen bonds [5].

When surrounding cells attach to a biomaterial surface, a series of physicochemical interactions occur between the cells and the surface. When a biomaterial implants (or is in a cell culture medium), protein adsorption occurs on the surface and mediates cell adhesion. At the same time, a signal is transmitted to the cell via intracellular adhesion receptors, especially integrins. Cells adhere to the surface and express active compounds for extracellular matrix deposition related to cell proliferation and differentiation. Adsorption of a protein to a biomaterial surface does not always increase cell adhesion. Tamada et al. determined that coating the surface with serum albumin reduces osteoblast adhesion while coating with fibronectin increases it [6]. Xu et al. reported that due to the amphiphilic properties of proteins, hydrophobic surfaces increase protein adsorption [7]. Related studies have shown that more cells adhere to hydrophilic surfaces. Keselowski et al. fabricated fibronectin-coated biomaterials and found that MC3T3 cells adhered to the surface containing the most hydroxyl groups. They also stated that cells adhered to surfaces containing the least methyl groups and attached to carboxylic acid and amine groups to the same extent [8]. According to Schmidt et al., a neutral or hydrophilic surface with methyl groups binds immunoglobulin G and fibronectin to stimulate leukocyte and phagocytic activity. They also stated that a positively charged and hydrophilic surface containing primary amine groups increased fibronectin binding and stimulated endothelial, myoblast, and osteoblast cell proliferation [9].

In the presented study, poly hydroxybutyrate-co-hexanoate (PHBHX) membranes were fabricated as antiadhesive patches for abdominal surgery. To prevent adhesion, the membranes were modified with oleic acid (OA), myristic acid (MA), polyethylene glycol 400 (PEG4), and polyethylene glycol 8000 (PEG8). The surface free energies and contact angles of the membranes were determined. The surface charges of the membranes were correlated with the cell adhesion and proliferation capacities. The effect of the membranes on blood coagulation factors was determined.

## 2. Material and methods

OA, MA, PEG4, PEG8, chloroform, human serum albumin (HSA), Coomassie blue G250, phosphoric acid, and dimethyl sulfoxide (DMSO) were purchased from Sigma (USA). Cell culture plates, trypsin-EDTA solution, and flasks were purchased from Gibco (USA). The PHBHX was donated by Kanaka (Japan).

### 2.1. Determination of the cytotoxicity of fatty acids and polyethylene glycols

Human fibroblast cells (primary dermal fibroblast, normal human adult (HDFa), ATCC PCS-201-012, USA) were cultured in 25 cm^2^ flasks in fibroblast medium (Fibroblast Growth Kit-Low Serum, ATCC PCS-201-041, USA) at 37 °C in a 5% carbon dioxide atmosphere. When the cells covered the flask surface, they were harvested with a Trypsin–EDTA solution (0.05% Trypsin, 0.02% EDTA).

The cytotoxicity of the additives (OA, MA, PEG4, and PEG8) on human fibroblast cells was determined according to ISO 10993-5 and ISO 10996-12 standards. For this purpose, the additives were diluted with cell culture medium at concentrations of 0.2, 0.1, 0.05, and 0.025 g/mL and sterilized with a 0.22 μm diameter injector filter. The fibroblasts were seeded into 96-well plates at a concentration of 1×10^4^ cells/mL. Diluted additives were pipetted onto the fibroblast cells. The standard medium was used as the negative control. A medium containing 20% DMSO was used as a positive control. After 24 h of incubation, cell viability was determined using an MTT assay [10].

### 2.2. Preparation of the membranes

For fabrication of the PHBHX membrane, 3 mL of 5% PHBHX (w/v) was dissolved in chloroform, and the solution was poured into glass molds. The solvent was evaporated for 3 days at room temperature. To fabricate modified membranes, 5% PHBHX (w/v) was dissolved in chloroform in a total volume of 3 mL. Aliquots of 50, 75, 250, and 500 μL of OA, MA, PEG4, or PEG8 were added to each PHBHX solution separately. After homogenization, the solutions were poured into glass molds. The solvent was evaporated for 3 days at room temperature to obtain the membranes. The nomenclature and contents of the membranes are given in Table 1.

### 2.3. Free surface energy and capacitance of the membranes

The contact angles and free surface energies of the membranes were determined with an optical contact angle measuring device (Attension Theta, Biolin Scientific, Sweden). The polar interaction values were calculated using the free surface energy values. Also, the capacitance values of the membranes were determined using a supercapacitor system (electrochemical double-layer capacitance). Capacitance is the ability of a component or circuit (in this case, the membranes) to collect and store energy in the form of an electrical charge. The capacitance values of the membranes were given as double-layer capacitance (pF) [11].

### 2.4 UV-Vis and ATR-FTIR studies

The homogeneity of the membranes was determined using solid UV-visible spectroscopy (Schimadzu, UV-2600). Modified membranes were placed in a solid UV device, and absorbance scanning was performed. A PHBHX membrane was used as a blank. Measurements were performed from three different points on each membrane, and the absorbance wavelengths were recorded. Chemical characterization of the membranes was determined using the ATR-FTIR device [12]. The studies were carried out with 16 measurements in the wavelength range of 600–4000 cm^−1^.

### 2.5. Thermal analysis of membranes

Thermal analyses of the membranes were done by thermogravimetric analysis (TGA, TA Instruments, Q600 SD) [13]. The analyses were performed with samples weighing 3–9 mg at temperatures of 25–450 °C, at increments of 10 °C.

### 2.6. Mechanical properties of membranes

The mechanical properties of the membranes were determined with a dynamic mechanical analyzer (Devotrans, GPUG/R, UK) [14]. The test speed was determined to be 10 mm/min, the preload (F0) was 0.01 N, and the preload speed was 2 mm/min. The approximate membrane length was 33 mm, and the approximate thickness was 0.6 mm.

### 2.7. Swelling and in vitro degradation

The gravimetric method was used to determine the swelling and degradation properties of the membranes [15]. The membranes were cut into squares of 1 cm in length and weighed for dry weight. Each membrane was placed in 5 mL of pH 7.4 phosphate buffer and incubated at 37 °C for 2 h. At the end of this period, the membranes were weighed for wet weight. The difference between dry and wet weight was calculated as the swelling rate.

For the degradation study, the membrane wet weight (incubated at 37 °C for 2 h) was considered 100%. The membranes were incubated at 37 °C for eight weeks and weighed once per week. The membrane weight changes over time were recorded, and the results were presented as degradation percentages.

### 2.8. Human serum albumin (HSA) absorption

The HSA absorption amounts of the membranes were determined. A 500 μg/mL concentration of HSA solution was prepared. The 1-cm^2^ membrane samples were incubated in HSA solution at 37 °C for 2 h, gently washed, and incubated in 250 μL of 0.5% Triton X100 solution for 30 min. Then, the amount of HSA in the solution was measured using the Coomassie blue reagent. The reagent was prepared by dissolving 10 mg of Coomassie blue G250 in 5 mL of 95% ethanol, adding 10 mL of 85% phosphoric acid, and filling to 100 mL with distilled water [16].

### 2.9. In vitro plasma coagulation factors

The effect of membranes on plasma coagulation factors was investigated. Membrane samples sized 5 mm^2^ were prepared. Rabbit blood was collected in citrate tubes and the tubes were centrifuged at 5000 rpm for 10 min. The plasma was collected and 250 μL of fresh plasma was pipetted onto the membranes. The samples were kept at 37 °C for 1 h [17]. At the end of the hour, prothrombin time (PTZ), activated partial thromboplastin time (aPTT), and fibrinogen amounts were measured (Sysmex, CA 600, Germany). Plasma that did not interact with a membrane was used as a control group.

### 2.10.Cell adhesion and proliferation studies

Human fibroblast cells were cultured on the membranes for 1, 3, and 5 days. The viability of the cells cultured on the membranes was determined using the MTT test at the end of the specified days [10].

### 2.11. Physical properties of membranes

The surface properties of some selected membranes (PHBHX, PHBHXMA3, PHBHXOA3, PHBHXPEG44, and PHBHXPEG83) were examined by scanning electron microscopy (SEM, Tescan, GAIA3+Oxford X-Max 150 EDS).

### 2.12. Statistical studies

Studies were carried out six times. Comparisons between groups were made using one-way analysis of variance and Tukey’s test (GraphPad Prism 9). A value of p < 0.05 was accepted as statistically significant.

## 3. Results and discussion

### 3.1. Determination of the cytotoxicity of the fatty acids and polyethylene glycols

**Figure 1:**
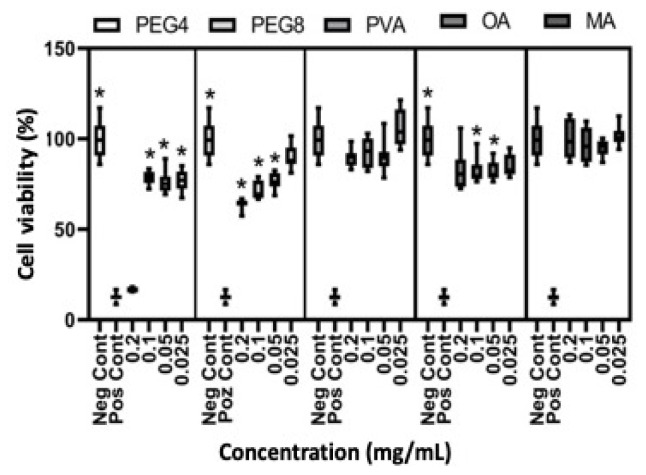
The cytotoxic effect of OA, MA, PEG4, and PEG8 on human fibroblast cells (n = 6, *p ≤ 0.05).

The cytotoxicity of OA, MA, PEG4, and PEG8 on HDFa cells was determined following ISO 10993-5 and ISO 10993-12 standards. The tests revealed that 0.2 g/mL concentrations of PEG4 and PEG8 were cytotoxic compared to the control (p < 0.05), but other fatty acids and diluted concentrations of polyethylene glycol were found to be not cytotoxic (p < 0.05) (Figure 1). Douglas et al. found that oleic acid reduced cytotoxicity in hamster fibroblast cells, which created oxygen cytotoxicity [18]. Cury-Boaventura et al. found no cytotoxicity after 24 and 48 h in the Raji cell line that interacted with 200μM OA. However, they determined that 25% OA was cytotoxic after 72 h [19]. The LD50 value of PEG4 on HeLa cells was 32.5 mg/mL and 24.7 mg/mL on L929 cells, according to Liu et al. [20].

### 3.2. Free surface energy and capacitance

**Figure 2:**
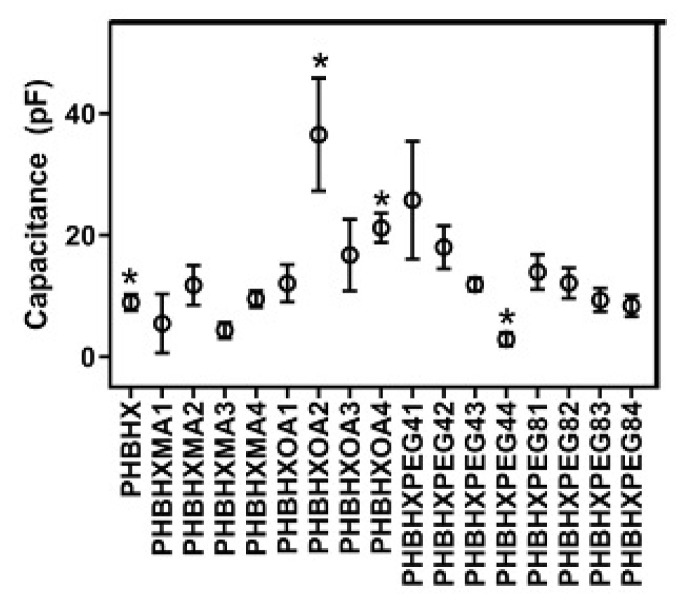
Capacitance values of the membranes (n = 6, *p ≤ 0.05).

The contact angle and free surface energies of the membranes against pure water, ethylene glycol, and diiodomethane were determined with an optical contact angle measuring device (Attension Theta, Biolin Scientific, Sweden) (Supplementary Figure 1). The percent polar interaction was calculated using the free surface energy. Results are given according to OWRK/Fowkes and Wu in Tables 2 and 3. It was observed that the OA modification increased the free surface energy (ytot), especially at high concentrations, and there was a fluctuation in the polar interaction. MA modification did not change the free surface energy (ytot), while a clear decrease was observed in the polar interaction. Depending on the concentration, both PEG4 and PEG8 reduced the contact angle value. There was no obvious change in the free surface energy (ytot). However, a significant decrease was observed in the polar interaction. The capacitances of the membranes were investigated, and it was seen that the capacitance values of high-dose PEG4 and MA-modified membranes were lower than the others (Figure 2). In the OA modification, the capacitance value fluctuated according to the concentration. The PHBHXOA4 membrane had the lowest percent polarity value.

### 3.3. Solid UV-Vis and ATR-FTIR studies

The homogeneity of the membranes was determined using solid UV-vis spectroscopy (Table 4). The wavelength of maximum absorbances of the membranes was similar, and it was predicted that OA, MA, PEG4, and PEG8 were homogeneously distributed in the membranes.

The chemical characterization of the membranes was determined by using the ATR-FTIR device. Studies were carried out with 16 measurements in the wavelength range of 600–4000 cm^−1^ (Supplementary Figures 2–5). An examination of the ATR-FTIR spectra of PHBHX membranes containing different ratios of OA revealed the C=O tensile bands of PHBHX at 1723 cm^−1^, asymmetric CH3 bending bands at 1460 cm^−1^, COO– asymmetric tensile bands at 1380 cm^−1^, and C–O–C tension bands at 1222 cm^−1^ [21]. Tensile bands of symmetrical and asymmetric –CH2 groups of OA at 2925 and 2855 cm^−1^, tensile bands of the C=O group at 1714 cm^−1^, C–O at 1280 cm^−1^, and OH at 980 cm^−1^ peaks were also observed [22,23]. The ATR-FTIR spectra of the PHBHX membranes containing different ratios of MA revealed the C=O tensile band of MA at 1723 cm^−1^. Symmetric and asymmetric tensile bands of the –CH2 group in the fatty acid were observed at 2916 and 2848 cm^−1^. The tensile peaks of the –OH group were seen at 686, 721, and 939 cm^−1^. The bending peak of the –CH2 group was observed at 1460 cm^−1^. Peaks of C–H vibrations were seen at 1280 cm^−1^ [24]. An examination of the ATR-FTIR spectra of PHBHX membranes containing different ratios of PEG4 revealed a band belonging to the –CH group of PEG at 980 cm^−1^. C–H bending in PEG was observed at 1280 cm^−1^, and a C–O–C ether tensile band was seen at 1094 cm^−1^ as well as at 2870–2950 cm^−1^ in bands corresponding to –C–H symmetric and asymmetric tensile vibrations. A –CH2 stretch band was seen at 2910 cm^−1^. A broad band of OH groups was observed at 3450 cm^−1^ [25]. The ATR-FTIR spectra of PHBHX membranes containing different ratios of PEG8 were examined, and a broad band of OH groups was seen at 3440 cm^−1^. Tensile peaks of C–H at 2875 cm^−1^ and C–O at 1100 cm^−1^ were observed. C–H deformation peaks were observed at 1465 cm^−1^ and 1380 cm^−1^. A band belonging to the –CH group of PEG was seen at 965 cm^−1^ [25,26].

### 3.4. Thermal analysis of membranes

Thermal analyses of the membranes were determined by TGA (Supplementary Figures 6–9). The membranes containing MA begin to lose mass at 230 °C and almost completely lost their mass by 275 °C without any gradual change. PHBHXMA1 lost mass gradually at 115 °C and 180 °C, losing 25% of its mass by 180 °C and almost all its mass by about 273 °C. PHBHXMA2 gradually lost mass at 115 °C and 185 °C, losing 37% of its mass by 185 °C and almost all of it by 273 °C. Gradual mass loss of PHBHXMA3 was observed at 115 °C and 215 °C. It lost approximately 67% of its mass by 215 °C and almost all of its mass by 282 °C. PHBHXMA4 gradually lost mass at 110 °C and 220 °C, losing approximately 77% of its mass by 220 °C and all of its mass by 273 °C. Thermal analyses of the PHBHX membranes containing different ratios of oleic acid revealed that PHBHXOA1 started to lose mass at 150 °C, losing 92% of its mass by 270 °C without gradual change. PHBHXOA2 started to lose mass at 150 °C, and lost approximately 84% of its mass at 270 °C. PHBHXOA3 started to lose mass at 150 °C, and lost approximately 79% of its mass at 270 °C. It gradually lost approximately 90% of its mass at 360 °C. PHBHXOA4 started to lose mass at 150 °C, losing approximately 75% of its mass at 285 °C and gradually losing approximately 97% of its mass by 380 °C. When thermal analyses of PHBHX membranes containing PEG400 in different ratios were examined, it was observed that PHBHXPEG41 gradually lost mass at 180 °C and 265 °C, losing about 82% of its mass at 265 °C and all of it at 360 °C. Gradual mass loss of PHBHXPEG42 was observed between 200 °C and 260 °C. It lost approximately 70% of its mass at 260 °C and 96% at 360 °C. PHBHXPEG43 was observed to lose mass gradually at 60 °C, 210 °C, and 265 °C. It lost 4% of its mass at 60 °C, about 55% at 265 °C, and almost all of its mass at 380 °C. PHBHXPEG44 was observed to lose mass gradually at 60 °C, 145 °C, 220 °C, and 260 °C. It lost 4% of its mass at 60 °C, about 55% at 260 °C, and almost all of its mass at 375 °C. When thermal analyses of the PHBHX membranes containing PEG8000 in different ratios were examined, it was observed that PHBHXPEG81 started to lose mass at 210 °C and lost its entire mass at 300 °C. Gradual mass loss was observed for PHBHXPEG82 at 210 °C and 285 °C, with approximately 97% of its mass lost at 285 °C and all of it lost at 300 °C. Gradual mass loss was observed at 210 °C and 294 °C for PHBHXPEG83. It lost approximately 70% of its mass at 294 °C and all of it at 410 °C. Gradual mass loss was observed at 185 °C, 232 °C, and 294 °C for PHBHXPEG84, and it lost approximately 55% of its mass at 290 °C and almost all of its mass at 420 °C.

### 3.5. Mechanical properties of membranes

The mechanical properties of the membranes were determined with a dynamic mechanical analyzer (Devotrans, GPUG/R, UK). The maximum force, maximum elongation, and maximum stress values of the membranes are given in Table 5. The breaking strength values of the PHBHX and PEG8-modified membranes were higher than the others. However, the MA and PEG4 modifications elongated the membrane more than the others. The stress at break and the thermal stability values of the PEG8-modified membranes were parallel. In a study with a human placenta, the breaking strength value of the placenta with a thickness of 43–305 μm was found as 0.8 ± 0.07 N, and the elongation at rupture was 17 ± 1% [27]. In a study with a human scalp, the tensile strength value of the left temporal skin was found as 3.42 N/mm^2^, and the elastic modulus value was 24.33 N/mm^2^. The tensile strength value of the right temporal skin was found to be 3.61 N/mm^2^, and the elastic modulus value was 25.2 N/mm^2^ [28]. However, Young’s modulus values for the transverse and longitudinal sections of the human abdominal wall are 0.0425 ± 0.009 and 0.0225 ± 0.0026 N/mm^2^, respectively [29]. In a study conducted with pigs, the rupture strength of the peritoneum was reported as between 0.525 ± 0.046 and 0.579 ± 0.178 N/mm^2^ according to gender. The rupture strength of the peritoneum on the right abdominal wall was reported as 4.530 0.855 N/mm^2^, and the rupture strength of the peritoneum above the right kidney was 3.609 0.182 N/mm^2^ [30].

### 3.6. Swelling and degradation properties of the membranes

**Figure 3:**
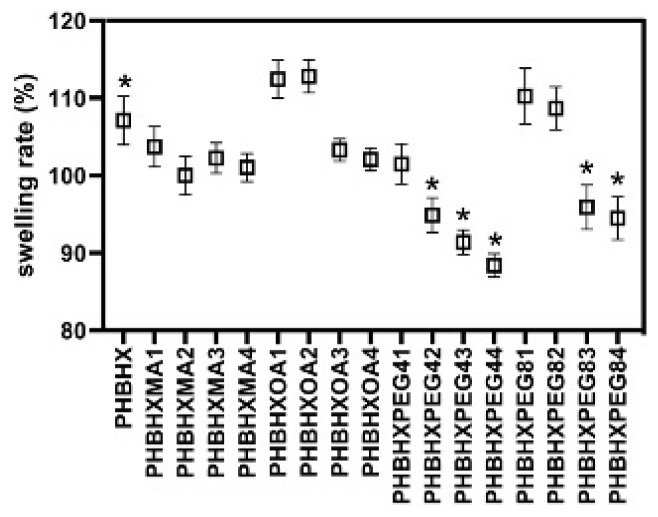
Swelling retention rate of the membranes (n = 6, *p ≤ 0.05).

The swelling and degradation properties of the membranes were determined by using the gravimetric method. The membranes retained water at rates of approximately 87.3%–110.8% (Figure 3). Low concentrations of OA and PEG8 led to greater water retention. There was no significant difference between the swelling ratio of PHBHX and the different ratios of the MA membranes (p > 0.05). Except for PHBHXPEG41, the swelling ratio of PEG4 formulations was lower than PHBHX (p < 0.05). Although PEG4 is hydrophilic, the swelling ratio of the membranes decreased as the amount of PEG4 in the formulation increased. This was interpreted as PEG4 being released. There was no significant difference between the PHBHX, PHBHXPEG81, and PHBHXPEG82 membranes (p > 0.05). However, there was a significant difference between the PHBHX, PHBHXPEG83 (p = 0.04), and PHBHXPEG84 membranes (p = 0.0095). This was interpreted as the membranes not releasing PEG8000 as their concentration increased (Supplementary Figures 10–13).

Comparing the PHBHX and MA-modified membranes, the wet weight of the PHBHX membranes decreased and deteriorated over time, and there was a significant difference between the results of the first and second weeks (p < 0.05). However, there was no significant difference between the degradation rates of the membranes comparing the second and eighth weeks (p > 0.05). Comparing the results of the first and second weeks, OA modification increased the rate of degradation with time (p < 0.05). In the second week, there was no significant difference between the MA and OA-modified membrane (p > 0.05). However, the degradation rate of PHBHXOA8 was higher than that of the other membranes in the sixth week (p < 0.05). There was a significant difference between PHBHX and PHBHXPEG44 in the second week (p < 0.05), when it was observed that the degradation of the PHBHXPEG44 was more rapid than and significantly different from the other PEG4 modifications (p < 0.05). The rapid degradation of the PHBHXPEG43 was significant (p < 0.05) by the fourth week. It was observed that the PHBHXPEG83 and PHBHXPEG84 membranes deteriorated much more than the other formulations in the second week (p < 0.05).

### 3.7. Human serum albumin (HSA) absorption

**Figure 4:**
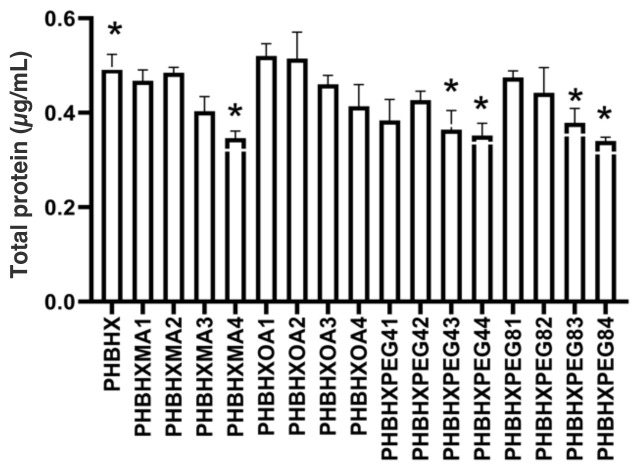
Human serum album adsorption of the membranes (n = 6, *p ≤ 0.05).

The HSA absorption of the membranes was measured with Coomassie blue (Figure 4). It was seen that PHBHXMA4 absorbed less protein than the bare PHBHX membrane (p < 0.05). However, there was no significant difference between the other MA-modified and the bare PHBHX membranes (p > 0.05). There was no significant difference between the OA-modified membranes and the bare PHBHX membranes (p > 0.05). PHBHXPEG4 (p < 0.05), PHBHXPEG83 (p < 0.05), and PHBHXPEG84 (p < 0.05) absorbed less albumin than bare PHBHX. High concentrations of MA reduced protein absorption. However, no effect of OA on protein absorption was observed. When PEG4 and PEG8 were compared, it was observed that polyethylene glycol modification decreased protein absorption, especially at the high-dose PEG8 modification, which decreased protein absorption more than the other modifications. The polar interaction value of PEG-modified membranes and the amount of protein absorbed were parallel. Contrary to the current findings, Zhang et al. fabricated a chitosan-PEG film and stated that PEG modification increased protein adsorption [31].

### 3.8. Plasma coagulation factors

**Figure 5:**
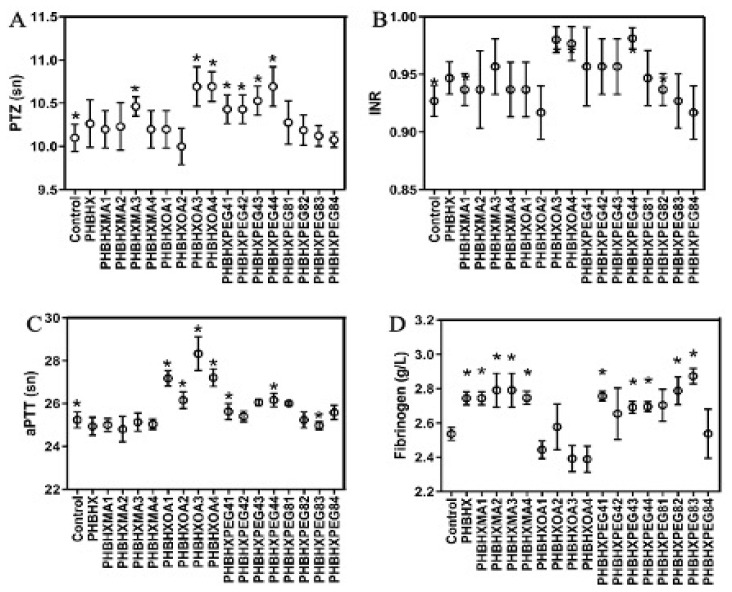
PTZ (A), INR (B), aPTT (C), and fibrinogen (D) results (n = 6, *p ≤ 0.05).

The effect of membranes on plasma coagulation factors was investigated. For this, square membranes sized 5 mm^2^ were set to interact with rabbit plasma. The PTZ values are shown in Figure 5A, and the international normalised ratio (INR) values are in Figure 5B. The PTZ of the PHBHX, PHBHXMA2, and PHBHXMA3 membranes was higher than in the control (p < 0.05). The PHBHXOA3 and PHBHXOA4 membranes increased the PTZ (p < 0.05), and all PEG4-modified and PHBHXPEG81 membranes had longer PTZ (p < 0.05). Examination of the INR values revealed that the INR values of the PHBHXOA3 and PHBHXPEG44 membranes increased when compared to the control (p < 0.05). Membranes of the other formulations did not have a significant effect on INR value (p > 0.05). The aPTT time in plasma that interacts with membranes is given in Figure 5C. It was observed that MA-modified membranes did not affect the plasma aPTT time (p > 0.05). On the other hand, PEG4, PEG8 modification, and especially OA significantly prolonged aPTT time (p < 0.05). The amounts of fibrinogen in the plasma that interact with membranes are given in Figure 5D. It was observed that a high concentration of OA modification (PHBHXOA3, PHBHXOA4) reduced fibrinogen value. The MA membrane modifications did not significantly change PTZ, PTT time, or fibrinogen values compared to the PHBHX membranes. However, a high concentration of OA increased the PTZ and PTT time and decreased the fibrinogen value compared to the PHBHX membranes. Likewise, PEG4-modified membranes increased the PTZ value but did not significantly change the PTT or fibrinogen. PEG8-modified membranes decreased the PTZ value but did not change the PTT, and high-dose PEG8 decreased the fibrinogen value. High concentrations of oleic acid (PHBHXOA3, PHBHXOA4) reduced fibrinogen amounts. Increased fibrinogen levels were observed in MA, PEG4, and PEG8. However, it is likely that the increased fibrinogen value was a meaningless cross-reaction effect. Thoistrup et al. described increased Factor VIIc activity in the extrinsic pathway of humans fed a diet rich in MA [32]. Benito et al. reported that people fed a diet rich in linoleic acid had no significant change in PTZ, aPTT, and antithrombin-3 levels [33]. Hoshi et al. reported that heparin-modified polytetrafluoroethylene (TEFLON) reduces blood coagulation and platelet adhesion [34].

### 3.9. Cell adhesion and proliferation

**Figure 6:**
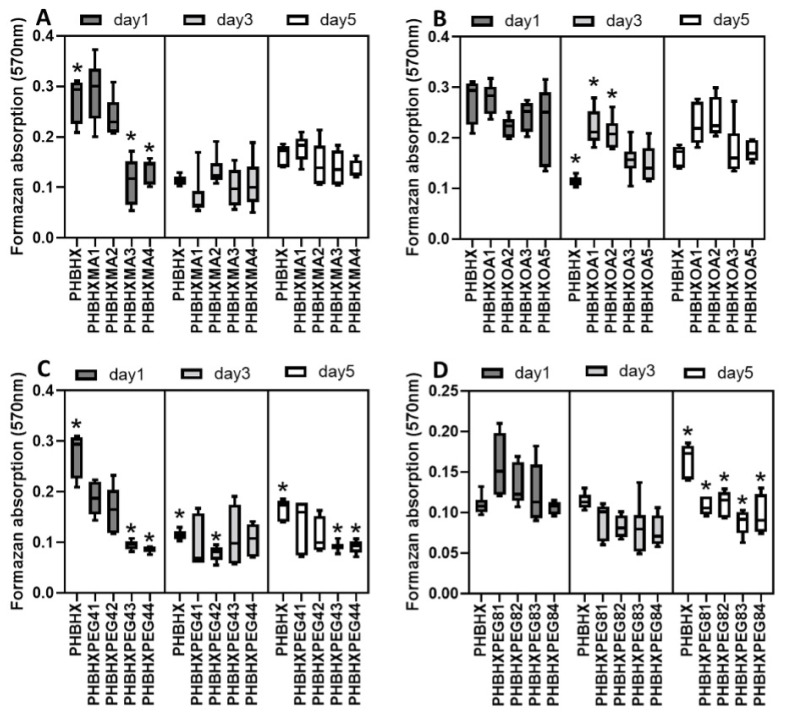
Human fibroblast attachment and proliferation on MA (A), OA (B), PEG4 (C), and PEG8 (D) modified membranes (n = 6, *p ≤ 0.05).

Human fibroblast cells were cultured on the membranes for 1, 3, and 5 days. At the end of the specified days, cell viability was determined by using the MTT test. After one day, it was seen that fewer cells adhered to the PHBHXMA3 and PHBHXMA4 membranes (Figure 6A) than to the PHBHX membranes (p < 0.05). No significant difference was observed between the membranes on the third day (p > 0.05). After the fifth day, fewer cells were cultured on the PHBHXMA4 membranes than the PHBHX (p < 0.05). A low concentration of MA did not affect cell adhesion or proliferation, while a high concentration reduced cell adhesion and proliferation. There was no significant difference between the OA-modified (Figure 6B) and PHBHX membranes (p > 0.05). Comparing the third and fifth days, more cells cultured on the PHBHXOA1 and PHBHXOA2 membranes than on PHBHX (p < 0.05). It was determined that OA modification at low concentrations increases cell proliferation. Magdalon et al. found that 50 μM oleic acid and linoleic acid stimulated cell proliferation, while palmitic acid decreased fibroblast proliferation [35]. Cell adhesion to the PEG4-modified membranes (Figure 6C) was less than that to PHBHX (p < 0.05). In the tests performed on the third day, there was no significant difference between the membranes (p > 0.05). However, after to the fifth day, fewer cells were cultured on PHBHXPEG43 and PHBHXPEG44 compared to the PHBHX membrane (p < 0.05). High-dose PEG4 reduced cell adhesion and proliferation. Cell adhesion to the PEG8-modified membranes (Figure 6D) was less than to PHBHX (p < 0.05). After the third day, no significant difference was observed between the membranes (p > 0.05). The concentration of cells cultured on the PEG-8-modified membranes was found to be lower than on the PHBHX membranes on the fifth day (p < 0.05), and PEG8 reduced cell adhesion and proliferation. Dahlin et al. reported lower proliferation of human gingival fibroblasts on PEG-modified surfaces compared to TCP plates [36]. Gupta et al. also found that PEG reduced the adhesion of human fibroblast cells [37].

### 3.10. Physical properties of membranes

**Figure 7:**
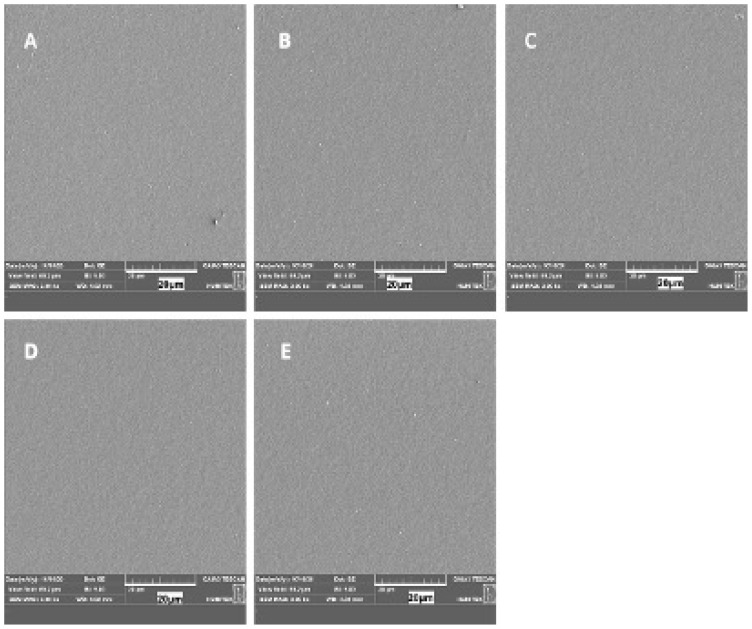
SEM images of the PHBHX (A), PHBHXMA3 (B), PHBHXOA3 (C), PHBHXPEG44 (D), and PHBHXPEG84 (E) membranes, magnitude ×3000.

Macroscopic photographs of all membranes (Supplementary Figure 14) and SEM images of selected formulations were taken. For the SEM studies, the membranes were adhered to the stubs and coated with 80-nm-thick gold palladium before being examined and photographed. Images of the formulations PHBHX, PHBHXMA3, PHBHXOA3, PHBHXPEG44, and PHBHXPEG83 are presented at 3000× magnification in Figure 7. The SEM photographs demonstrate that all formulations had a flat, smooth surface.

## 4. Conclusion

MA slightly increased surface free energy and did not affect cell adhesion or proliferation at low concentrations, but it did at high concentrations. The decrease in cell proliferation was due to MA decreasing the value of the membrane–polar interaction. In addition, MA made the membrane stick to the cell culture dish and reduced the processability of the membrane. Similarly, it was observed that modification with OA slightly increased the surface free energy and did not cause adhesion to the cell culture dish. Modification with oleic acid did not affect the polar interaction of the membranes and increased cell proliferation at low concentrations. PEG4 and PEG8 decreased cell adhesion and proliferation. The PEG4 and PEG8 modifications did not change the surface free energy of the membranes but decreased the percentage value of the polar interaction. It is suggested that the reason for the modification of PEG4 and PEG8 to reduce cell proliferation is to reduce the percent polar interaction.

## Supplementary Figures

Supplementary Figure 1 Contact angle images of the membranes versus pure water.

Supplementary Figure 2 ATR-FTIR spectra of the PHBHX membranes containing OA.

Supplementary Figure 3 ATR-FTIR spectra of the PHBHX membranes containing MA.

Supplementary Figure 4 ATR-FTIR spectra of the PHBHX membranes containing PEG4.

Supplementary Figure 5 ATR-FTIR spectra of the PHBHX membranes containing PEG8.

Supplementary Figure 6 Thermal analysis of the PHBHX membranes containing MA.

Supplementary Figure 7 Thermal analysis of the PHBHX membranes containing OA.

Supplementary Figure 8 Thermal analysis of the PHBHX membranes containing PEG4.

Supplementary Figure 9 Thermal analysis of the PHBHX membranes containing PEG8.

Supplementary Figure 10 Degradation rates of the PHBHX and PHBHXMA membranes.

Supplementary Figure 11 Degradation rates of the PHBHXOA membranes.

Supplementary Figure 12 Degradation rates of the PHBHXPEG4 membranes.

Supplementary Figure 13 Degradation rates of the PHBHXPEG8 membranes.

Supplementary Figure 14 Macroscopic photographs of the membranes.

## Figures and Tables

**Table 1:** Nomenclature and contents of the membranes.

	PHBHX (g)	MA (μL)	OA (μL)	PEG4 (μL)	PEG8 (μL)	Chloroform (mL)
PHBHX	0.15	-	-	-	-	3
PHBHXMA1	0.15	50	-	-	-	2.95
PHBHXMA2	0.15	75	-	-	-	2.925
PHBHXMA3	0.15	250	-	-	-	2.75
PHBHXMA4	0.15	500	-	-	-	2.5
PHBHXOA1	0.15	-	50	-	-	2.95
PHBHXOA2	0.15	-	75	-	-	2.925
PHBHXOA3	0.15	-	250	-	-	2.75
PHBHXOA4	0.15	-	500	-	-	2.5
PHBHXPEG41	0.15	-	-	50	-	2.95
PHBHXPEG42	0.15	-	-	75	-	2.925
PHBHXPEG43	0.15	-	-	250	-	2.75
PHBHXPEG44	0.15	-	-	500	-	2.5
PHBHXPEG81	0.15	-	-	-	50	2.95
PHBHXPEG82	0.15	-	-	-	75	2.925
PHBHXPEG83	0.15	-	-	-	250	2.75
PHBHXPEG84	0.15	-	-	-	500	2.5

**Table 2:** Free surface energy, polar interaction, and contact angle values of the membranes modified with MA or OA.

	OWRK/Fowkes				Wu				Contact angles
	γtot (mN/m)	γd (mN/m)	γp (mN/m)	Polar interaction (%)	γtot (mN/m)	γd (mN/m)	γp (mN/m)	Polar interaction (%)	
Myristic acid-modified membranes									
PHBHX	39.1±6.3	34.4±5.5	4.6±0.7	11.9±1.9	41.9±6.7	33.9±5.4	8.1±13	19.2±3.1	73.9±10.8
PHBHXMA1	38.4±4.6	28.3±3.4	10.2±1.0	26.5±3.2	41.8±5.0	27.4±3.3	14.4±1.7	34.5±4.1	55.3±6.6
PHBHXMA2	38.4±4.2	33.8±3.7	4.6±0.5	11.9±1.3	41.3±4.5	33.4±3.7	7.9±0.9	19.3±2.1	73.3±8.1
PHBHXMA3	38.5±3.9	36.9±3.7	1.7±0.2	4.3±0.4	40.8±4.1	36.4±3.7	4.4±0.4	13.1±1.3	78.3±7.9
PHBHXMA4	40.7±4.2	38±3.9	2.7±0.3	6.7±0.7	43.4±4.5	37.7±3.9	5.7±0.6	10.8±1.1	86.5±6.9
Oleic acid-modified membranes									
PHBHX	39.08±4.1	34.43±3.6	4.64±0.5	11.88±1.1	41.98±3.4	33.93±3.6	8.05±0.6	19.17±1.0	73.97±6.8
PHBHXOA1	42.94±4.4	27.99±2.9	14.95±1.5	34.81±3.6	46.56±3.7	27.26±2.8	19.31±1.0	41.46±3.2	52.97±4.4
PHBHXOA2	44.66±4.7	34.77±3.7	9.89±1.0l	22.16±2.1	47.54±4.0	33.34±2.5	14.21±0.1	29.88±2.2	61.54±5.5
PHBHXOA3	43±4.4	35.86±3.7	7.14±0.7	16.6±1.7	45.94±3.7	34.81±2.6	11.12±0.6	24.21±1.5	67.44±5.9
PHBHXOA4	45.91±4.8	40.68±4.0	5.23±0.5	11.4±0.9	48.53±4.0	39.14±3.1	9.4±0.7	19.36±1.0	70.73±6.4

**Table 3:** Free surface energy, polar interaction, and contact angle values of the membranes modified with PEG4 or PEG8.

	OWRK/Fowkes				Wu				Contact angles
	γtot (mN/m)	γd (mN/m)	γp (mN/m)	Polar interaction (%)	γtot (mN/m)	γd (mN/m)	γp (mN/m)	Polar interaction (%)	
Membranes modified with PEG4									
PHBHX	39.1±2.1	34.4±2.2	4.6±0.7	11.9±1.1	41.9±3.4	33.9±2.1	8.05±0.6	19.2±0.6	73.9±8.8
PHBHXPEG41	43.2±3.2	36.5±3.2	6.7±0.5	15.6±2.6	46.1±4.1	35.3±1.6	10.7±0.7	23.3±1.1	70.1±6.4
PHBHXPEG42	42.8±1.9	37.8±3.5	4.9±0.3	11.6±1.8	45.6±3.7	36.8±2.2	8.8±0.2	19.3±0.8	68.5±5.1
PHBHXPEG43	40.2±3.5	37.5±2.9	5.6±0.3	14.3±2.3	42.4±3.8	36.3±2.4	9.3±0.1	21.9±1.2	69.6±3.4
PHBHXPEG44	39.6±2.6	33.9±1.9	2.7±1.5	6.6±0.3	42.4±2.8	33.1±1.4	6.1±0.1	14.4±0.9	66.3±9.6
Membranes modified with PEG8									
PHBHX	39.1±3.3	34.4±2.6	4.6±0.7	11.9±1.5	41.9±3.1	33.9±1.4	8.1±0.2	19.2±2.1	73.9±7.5
PHBHXPEG81	42.4±2.8	37.5±3.2	4.9±0.1	11.6±1.1	45.2±2.6	36.5±2.2	8.7±0.3	19.2±1.1	70.3±6.8
PHBHXPEG82	42.5±3.6	34.1±3.1	8.4±0.2	19.8±1.3	45.4±1.1	32.6±2.6	12.8±0.6	28.1±2.7	64.9±3.5
PHBHXPEG83	40.3±2.7	33.2±1.7	7.1±0.6	17.6±1.1	43.2±2.4	32.3±3.0	10.9±0.2	25.2±3.1	58.5±2.3
PHBHXPEG84	37.8±1.8	35.8±2.6	1.9±0.1	5.1±0.2	40.3±3.8	35.7±2.1	4.6±0.1	11.3±0.8	60±1.2

**Table 4:** Maximum absorbance values obtained from solid UV-Vis absorbance scanning of the membranes.

Membrane formulation	Maximum absorbance	Membrane formulation	Maximum absorbance
PHBHXMA1	223	PHBHXOA3	285
PHBHXMA2	223	PHBHXOA4	284
PHBHXMA3	224	PHBHXPEG41	220
PHBHXMA4	223	PHBHXPEG42	225
PHBHXOA1	242	PHBHXPEG43	218
PHBHXOA2	247	PHBHXPEG44	220

**Table 5:** Tensile test result of the membranes.

	Fmax force (N)	Fmax elongation Δl max (mm)	Fmax elongation ɛmax (%)	Max stress σZB (N/mm^2^)
PHBHX	7.105	1.949	5.906	11.842
PHBHXMA1	3.114	1.577	4.779	5.19
PHBHXMA2	2.187	1.001	3.033	3.645
PHBHXMA3	1.848	2.816	8.533	3.08
PHBHXMA4	1.246	2.948	8.933	2.077
PHBHXOA1	3.169	1.22	3.697	5.282
PHBHXOA2	2.774	2.044	6.194	4.623
PHBHXOA3	1.587	1.672	5.067	2.645
PHBHXOA4	0.863	2.379	7.209	1.438
PHBHXPEG41	1.859	9.128	27.661	3.098
PHBHXPEG42	2.663	3.647	11.052	4.438
PHBHXPEG43	2.091	2.219	6.724	3.485
PHBHXPEG44	1.107	2.902	8.794	1.845
PHBHXPEG81	5.444	1.33	4.03	9.073
PHBHXPEG82	2.977	0.907	2.748	4.962
PHBHXPEG83	6.648	1.329	4.027	11.08
PHBHXPEG84	7.197	0.834	2.527	11.995
