# Supplementary figures and images for: Nonadhesive membranes: preparation and characterization of modified PHBHX membranes
Source: Turk J Chem. 2024 Nov 20;49(1):54–67. doi: 10.55730/1300-0527.3710 (PMC11913361; doi:10.55730/1300-0527.3710)

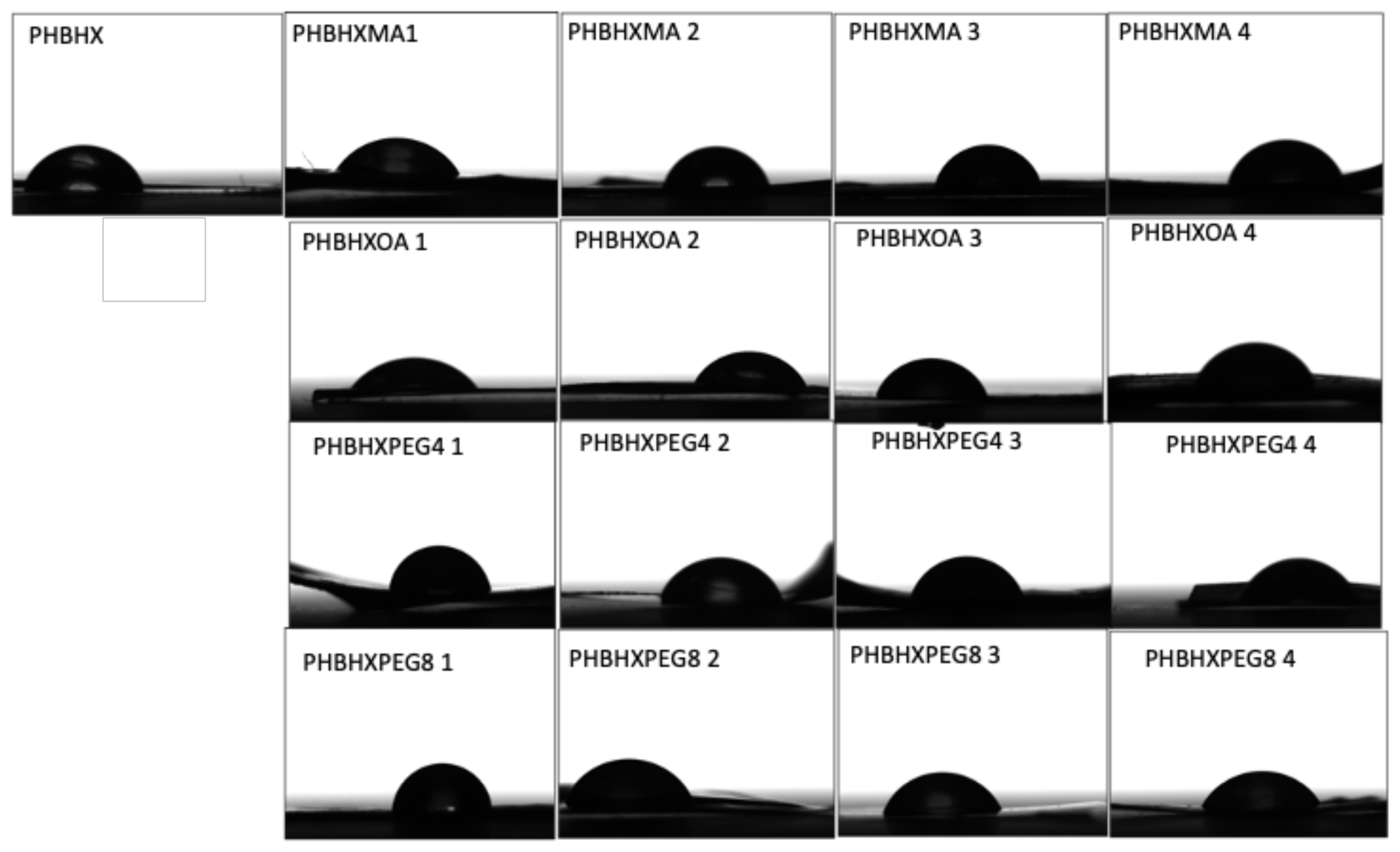

Supplement: Supplementary Figure 1 — Contact angle images of the membranes versus pure water. [file tjc-49-01-54s1.tif]

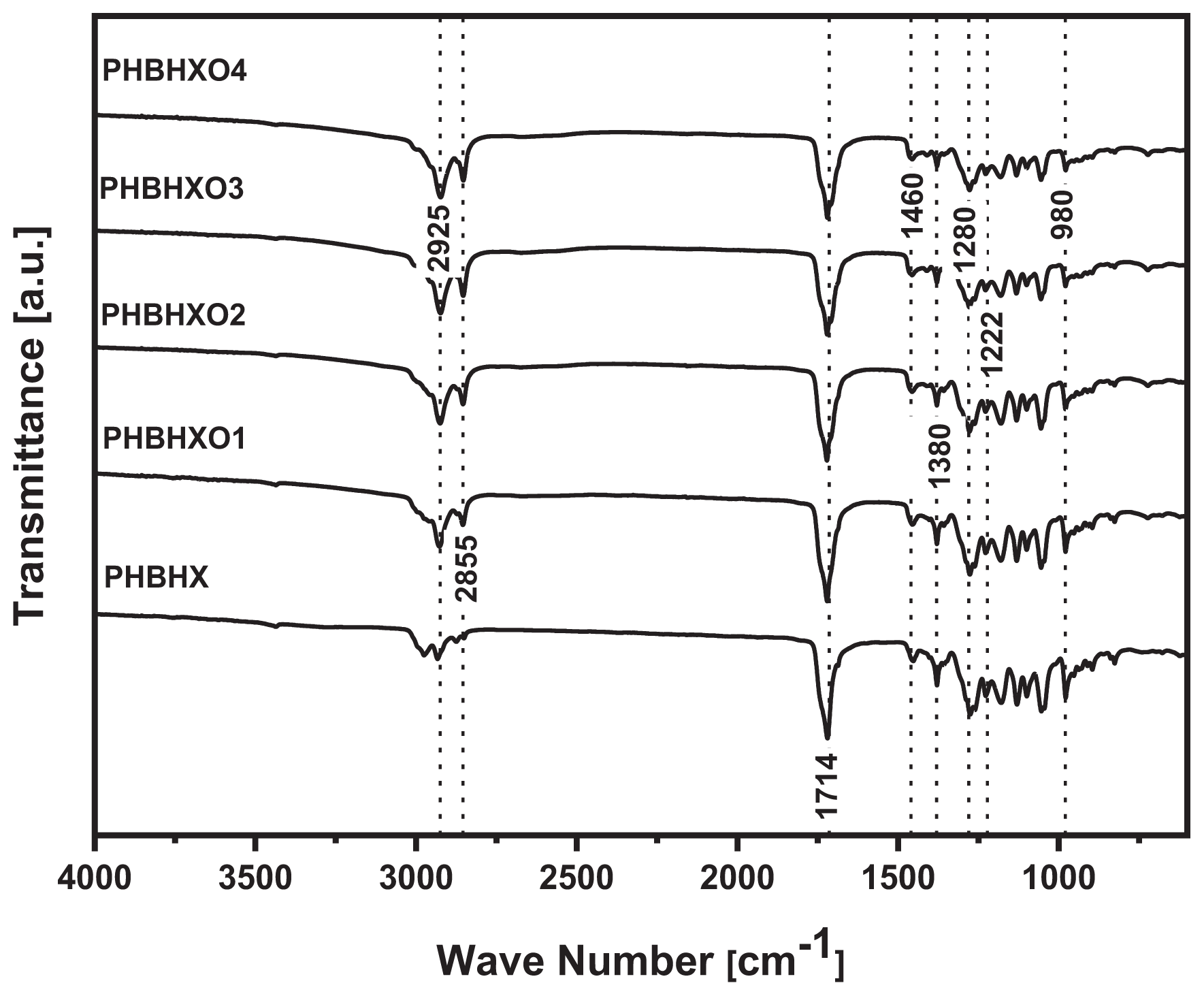

Supplement: Supplementary Figure 2 — ATR-FTIR spectra of the PHBHX membranes containing OA. [file tjc-49-01-54s2.tif]

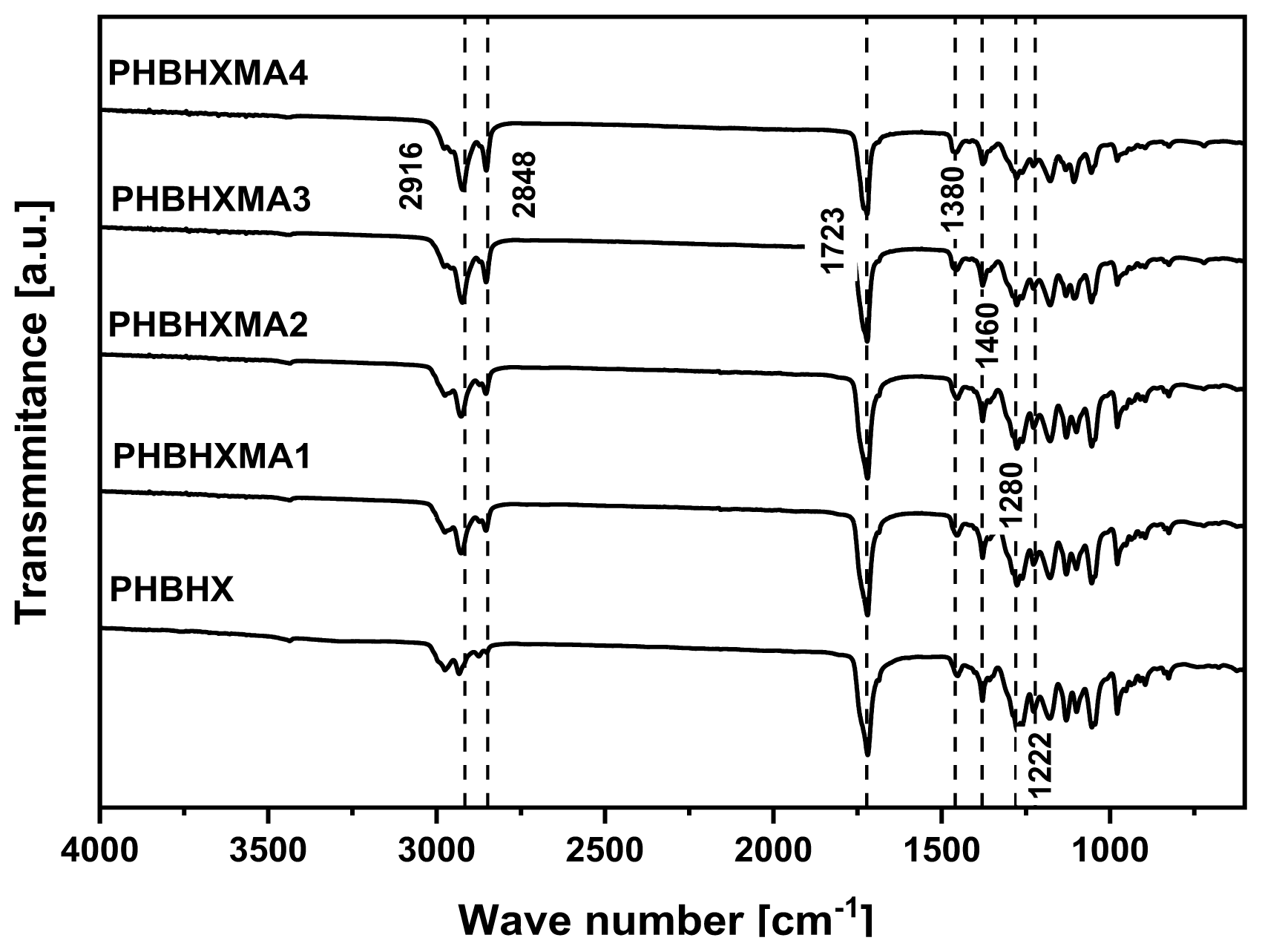

Supplement: Supplementary Figure 3 — ATR-FTIR spectra of the PHBHX membranes containing MA. [file tjc-49-01-54s3.tif]

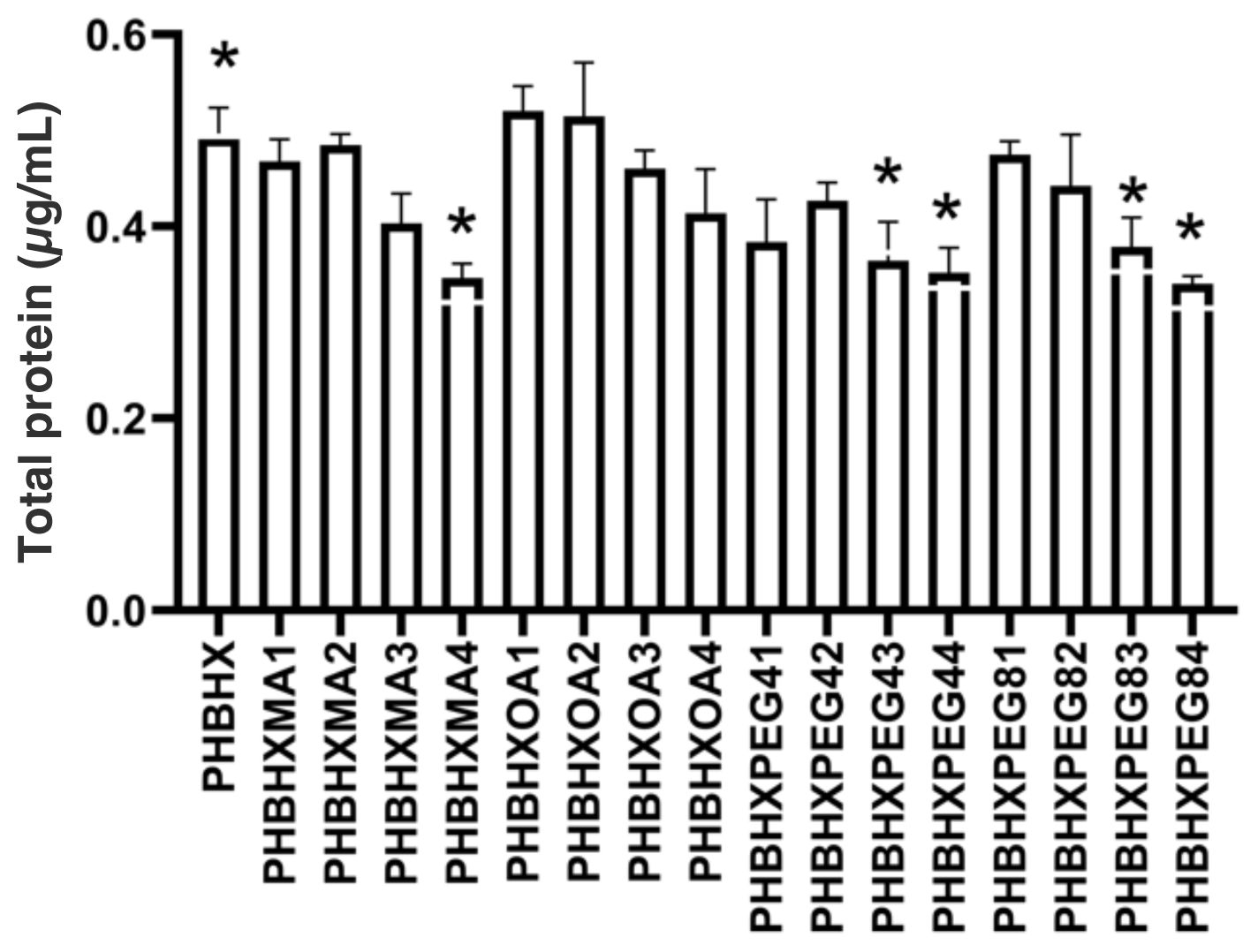

Supplement: Supplementary Figure 4 — ATR-FTIR spectra of the PHBHX membranes containing PEG4. [file tjc-49-01-54s4.tif]

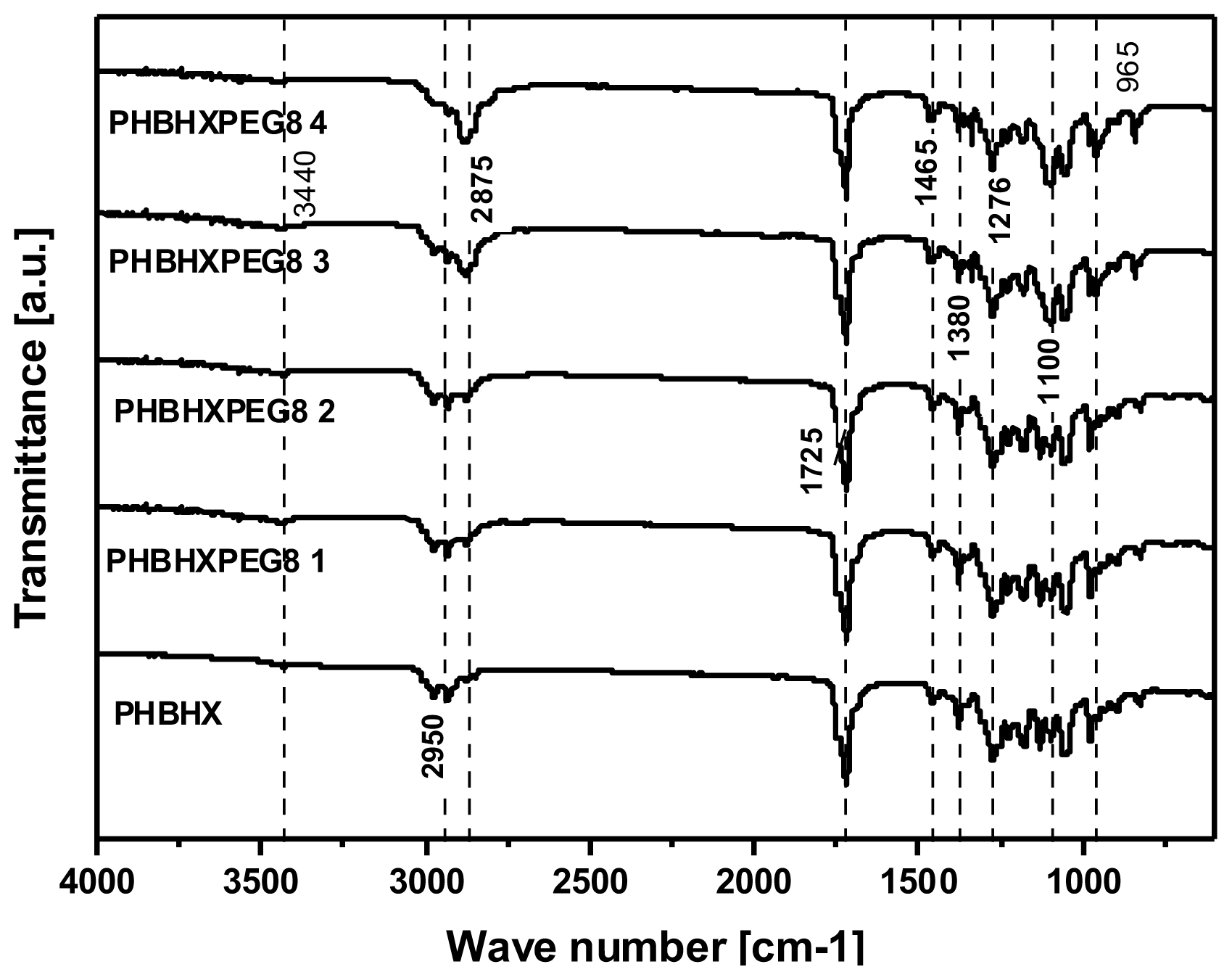

Supplement: Supplementary Figure 5 — ATR-FTIR spectra of the PHBHX membranes containing PEG8. [file tjc-49-01-54s5.tif]

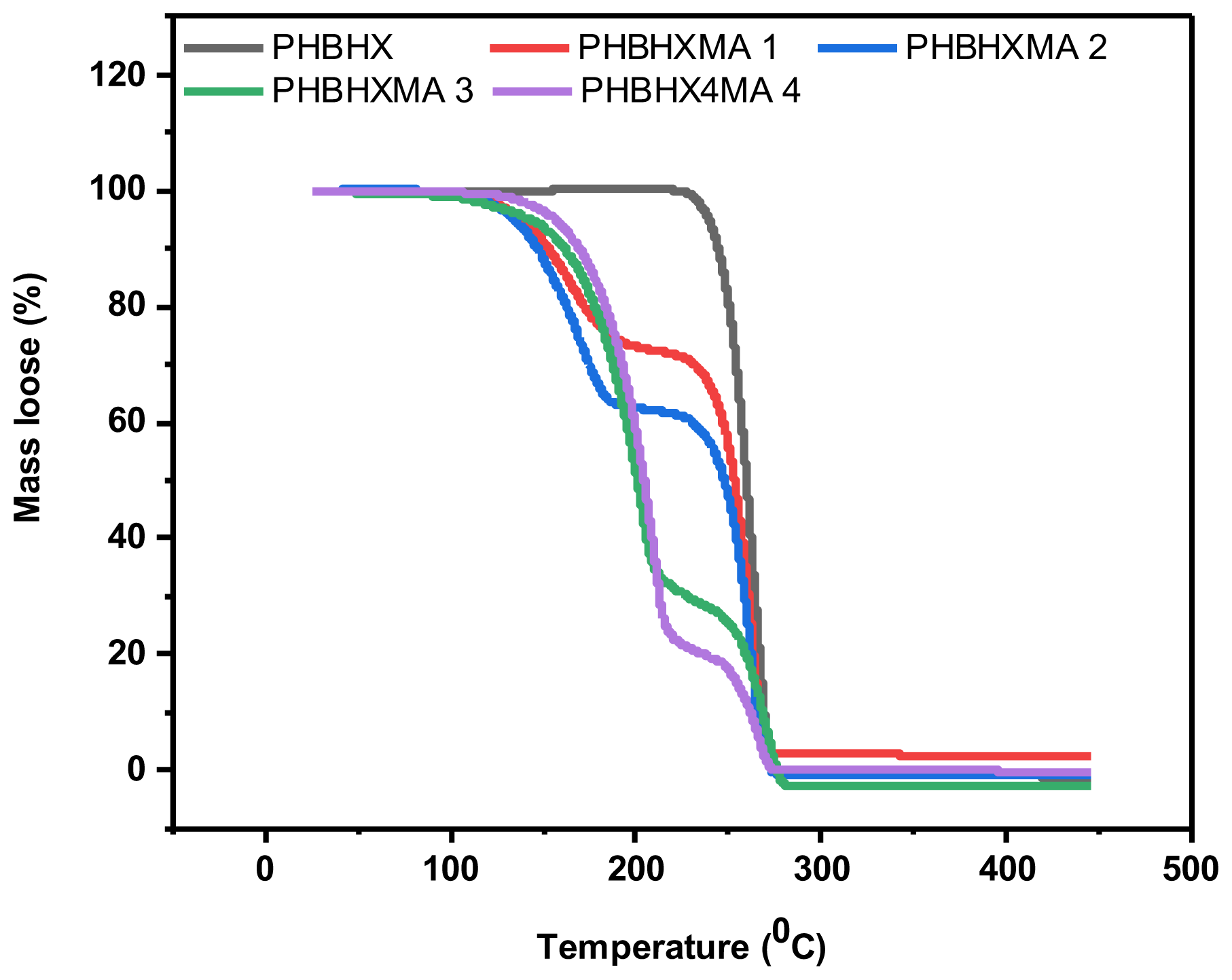

Supplement: Supplementary Figure 6 — Thermal analysis of the PHBHX membranes containing MA. [file tjc-49-01-54s6.tif]

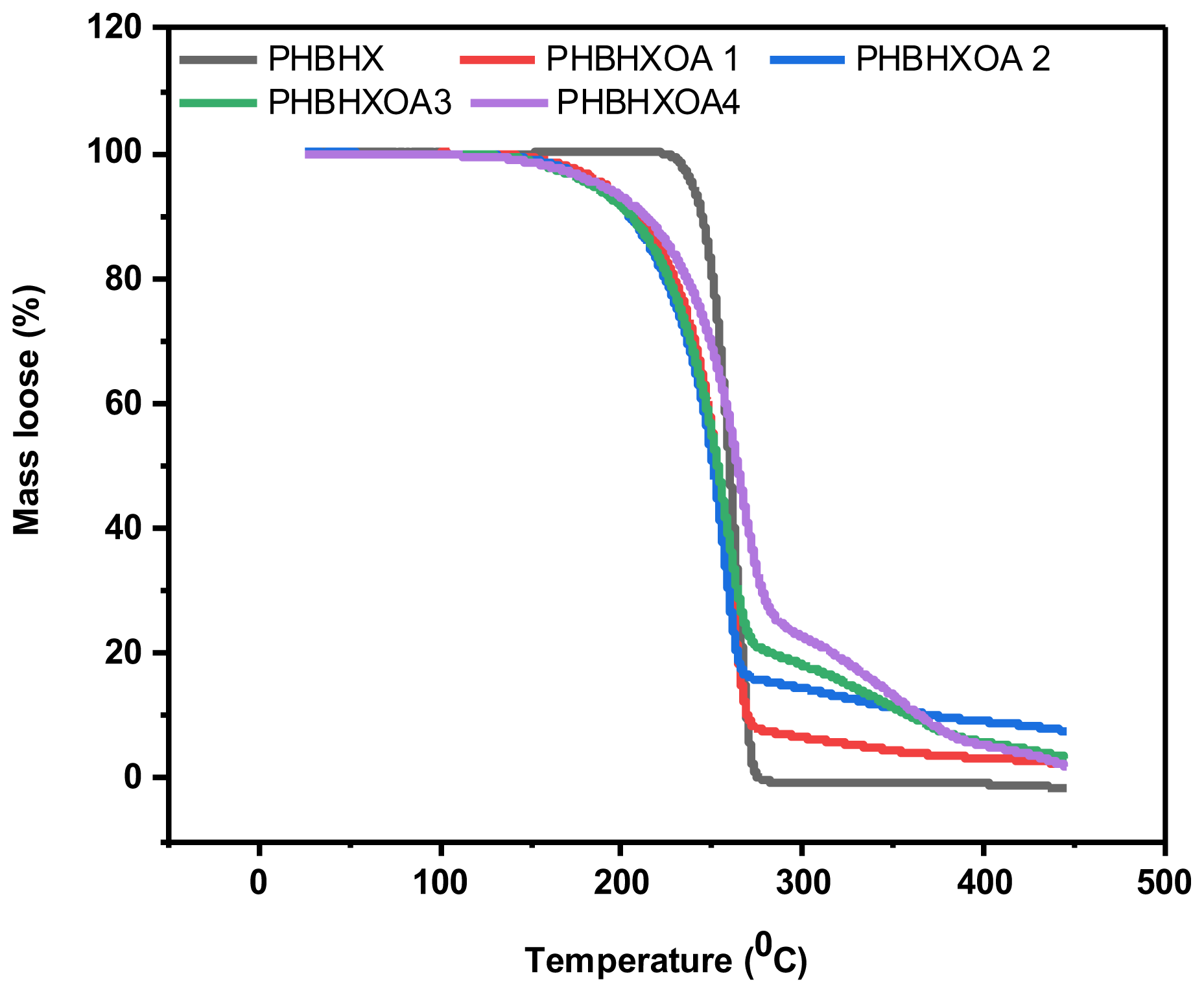

Supplement: Supplementary Figure 7 — Thermal analysis of the PHBHX membranes containing OA. [file tjc-49-01-54s7.tif]

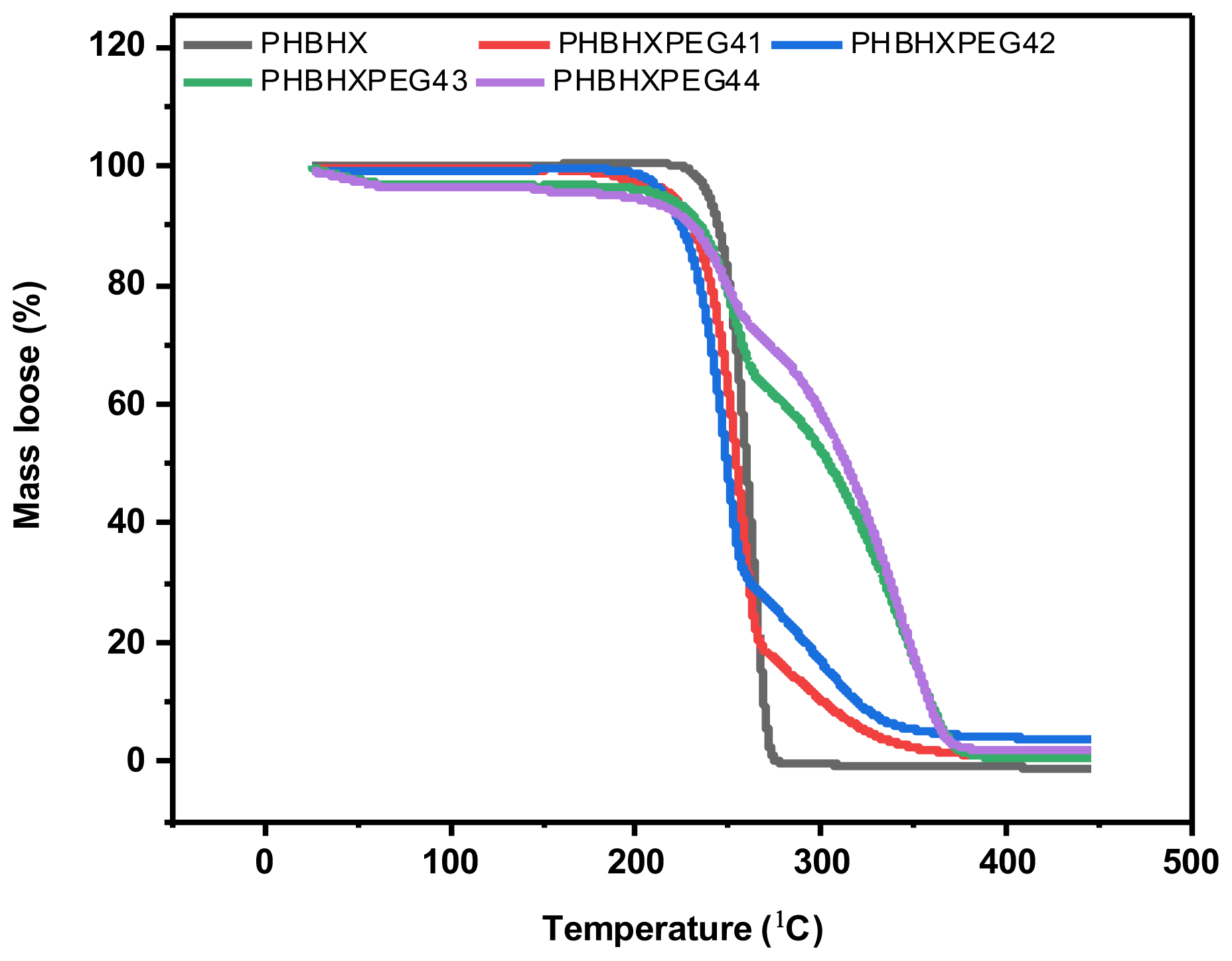

Supplement: Supplementary Figure 8 — Thermal analysis of the PHBHX membranes containing PEG4. [file tjc-49-01-54s8.tif]

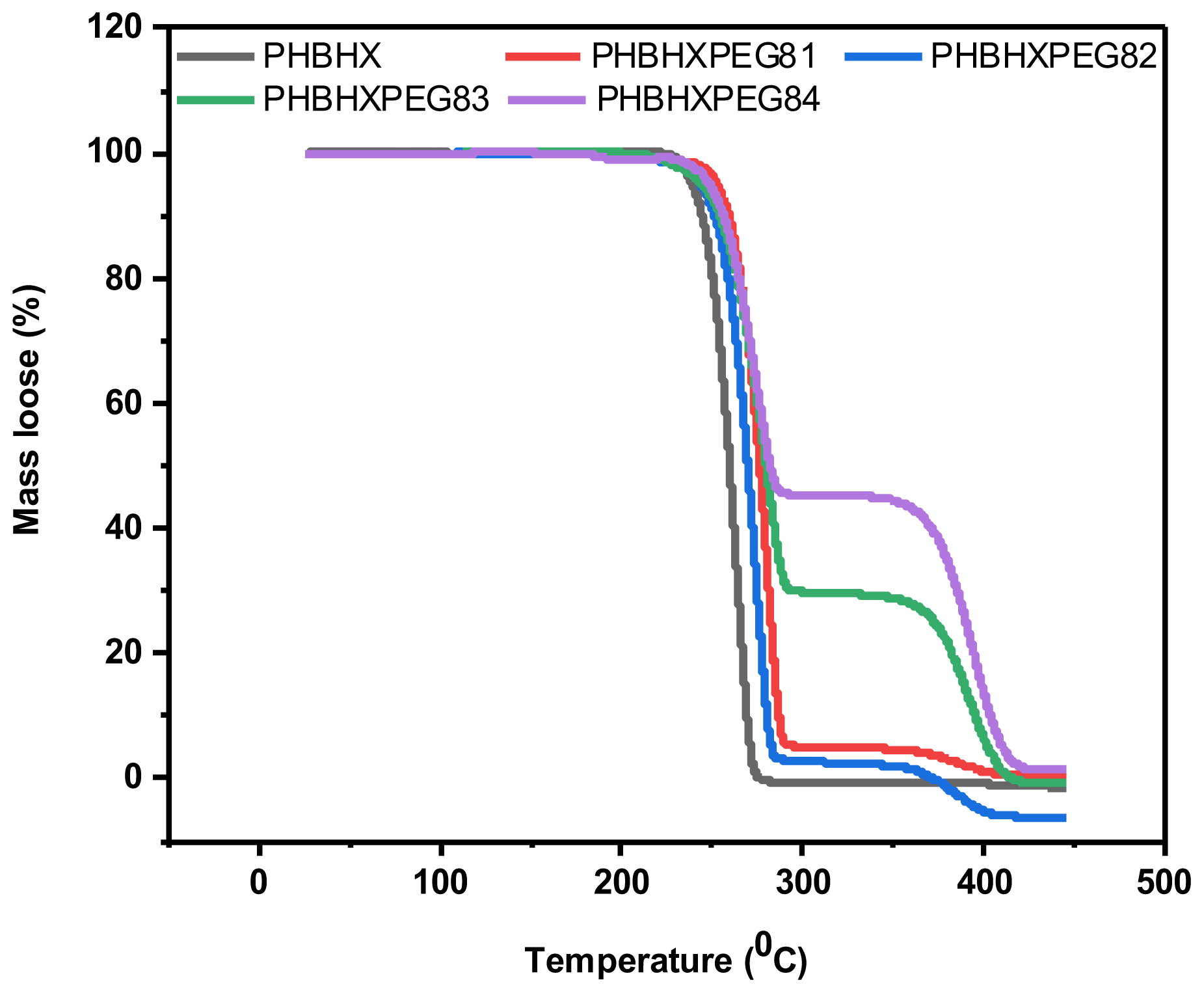

Supplement: Supplementary Figure 9 — Thermal analysis of the PHBHX membranes containing PEG8. [file tjc-49-01-54s9.tif]

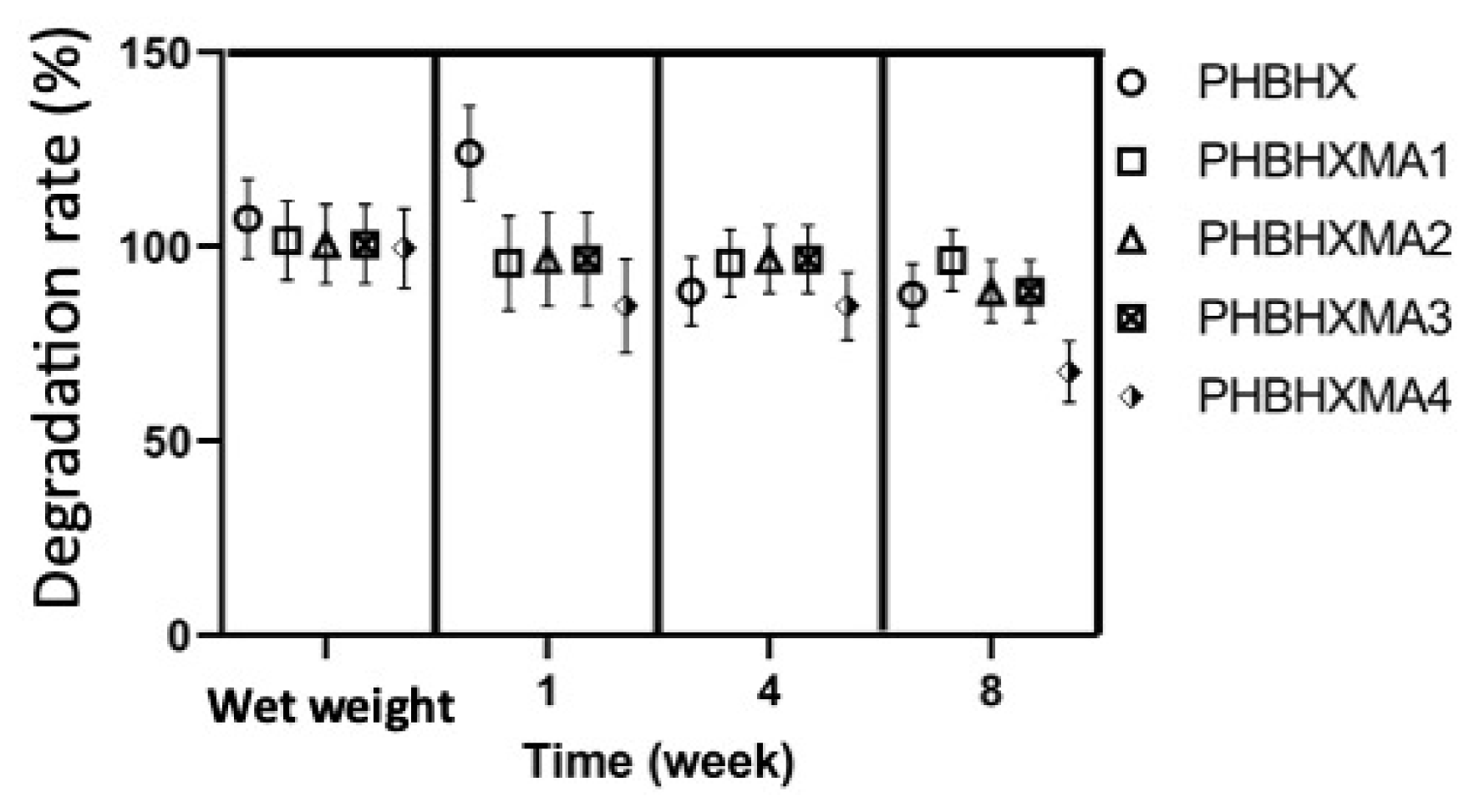

Supplement: Supplementary Figure 10 — Degradation rates of the PHBHX and PHBHXMA membranes. [file tjc-49-01-54s10.tif]

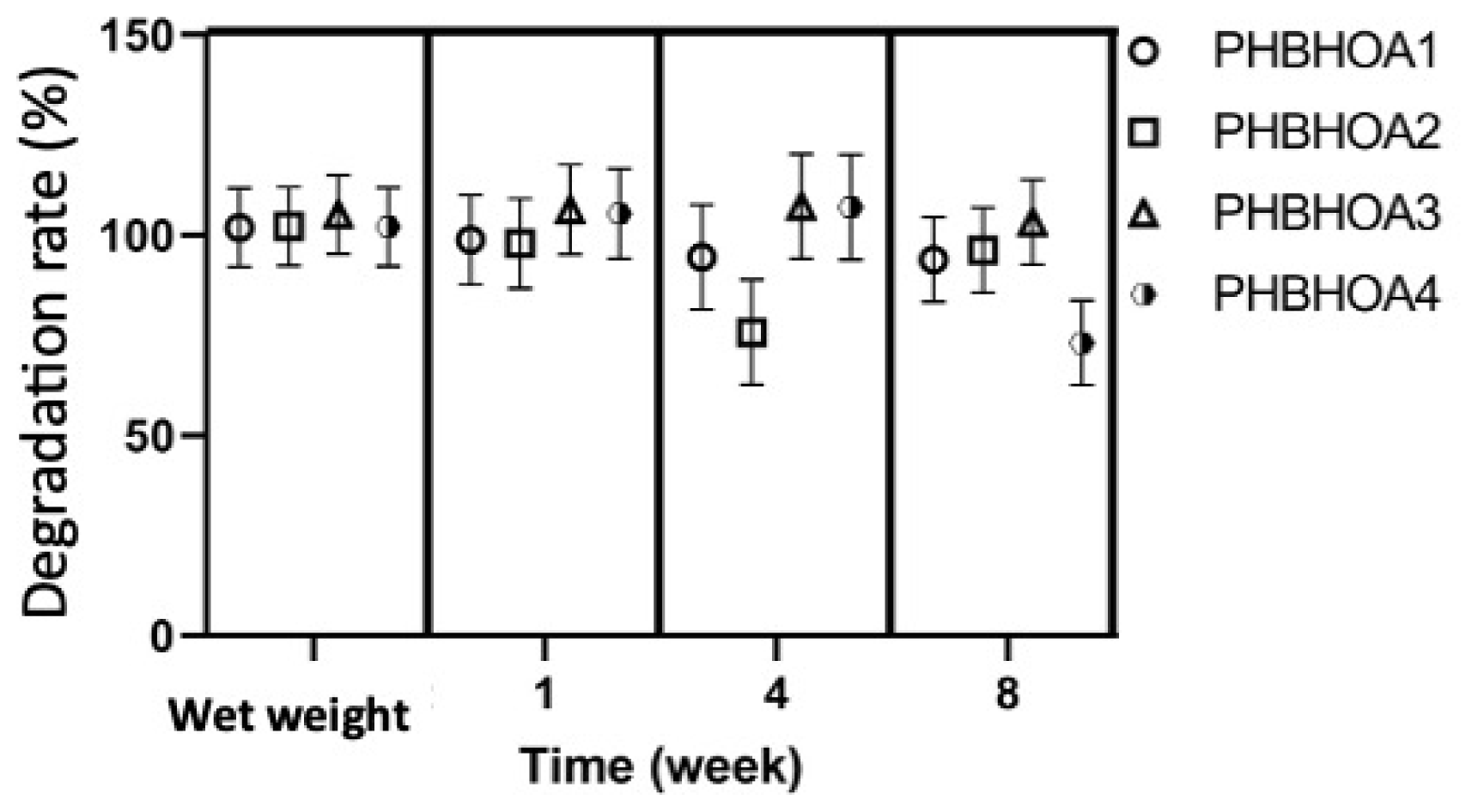

Supplement: Supplementary Figure 11 — Degradation rates of the PHBHXOA membranes. [file tjc-49-01-54s11.tif]

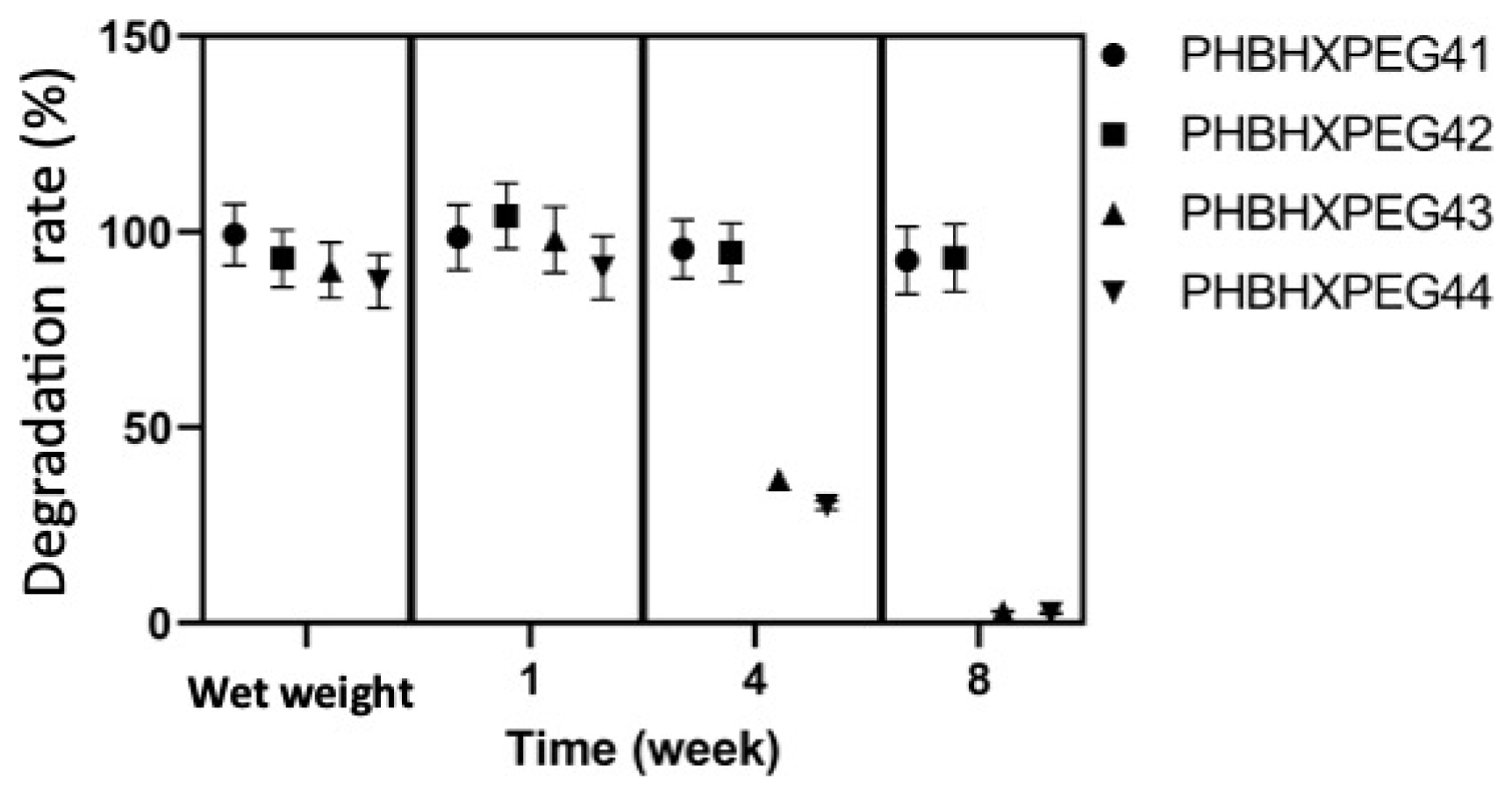

Supplement: Supplementary Figure 12 — Degradation rates of the PHBHXPEG4 membranes. [file tjc-49-01-54s12.tif]

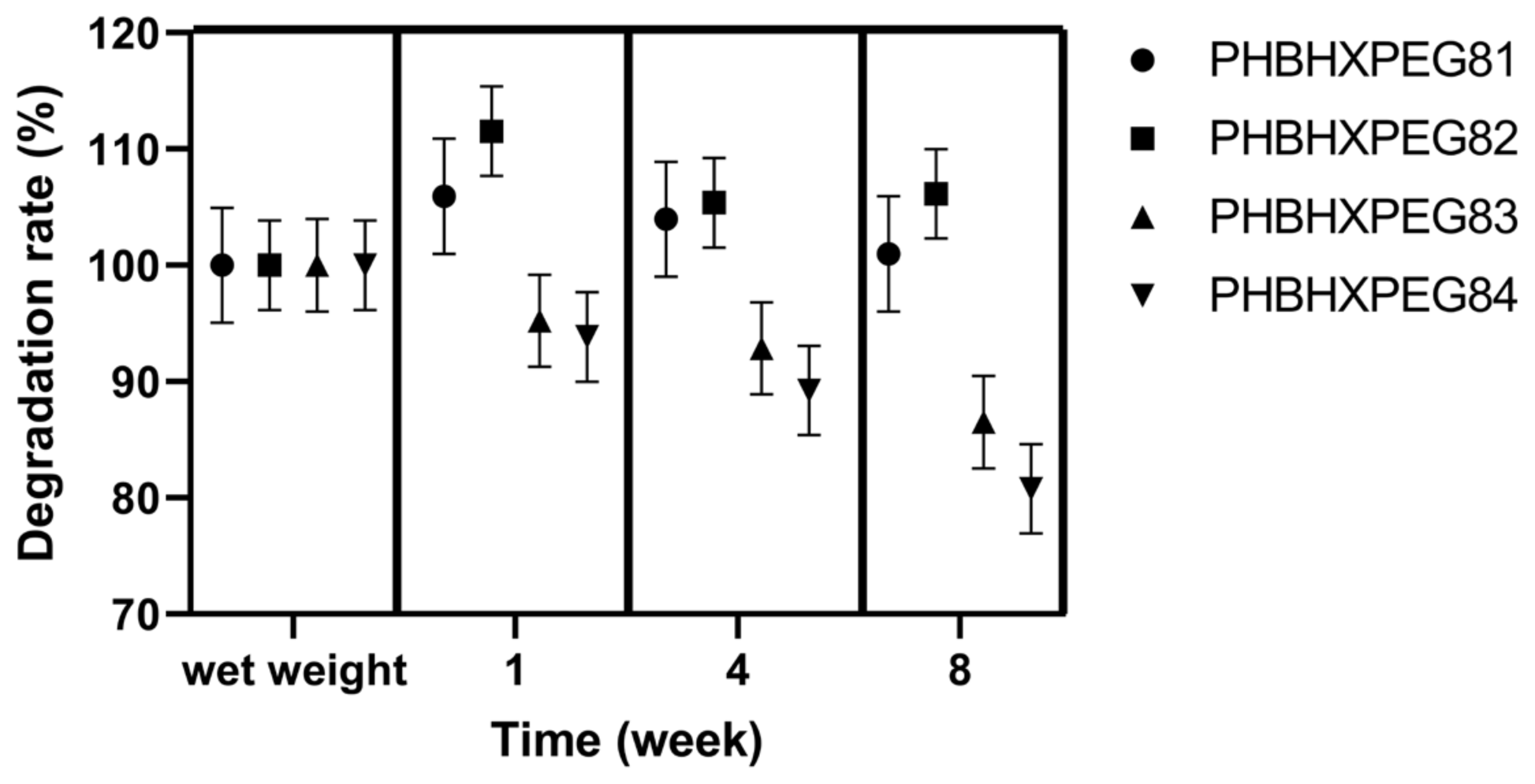

Supplement: Supplementary Figure 13 — Degradation rates of the PHBHXPEG8 membranes. [file tjc-49-01-54s13.tif]

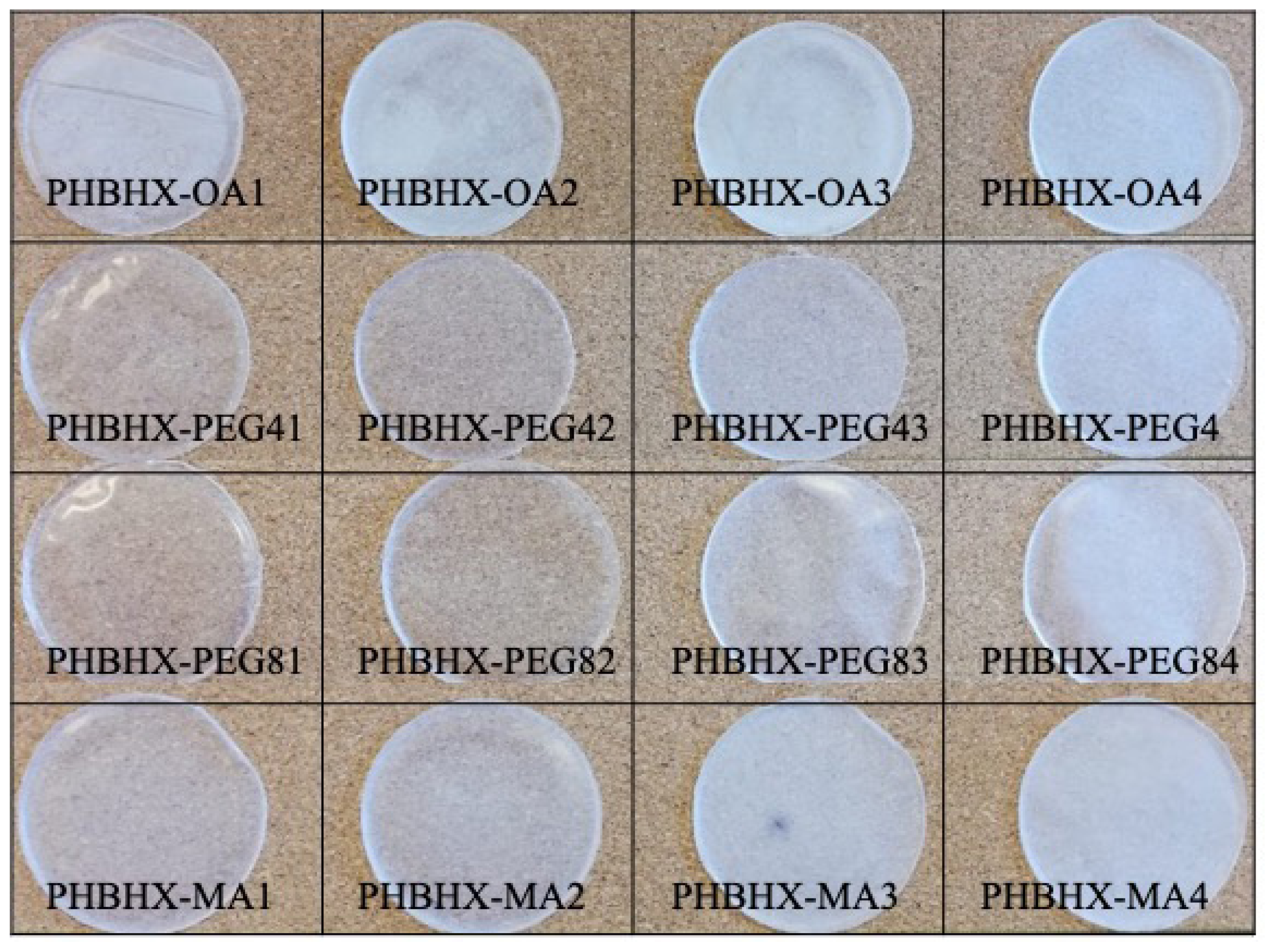

Supplement: Supplementary Figure 14 — Macroscopic photographs of the membranes. [file tjc-49-01-54s14.tif]
